# Anti-Protease Activity Deficient Secretory Leukocyte Protease Inhibitor (SLPI) Exerts Cardioprotective Effect against Myocardial Ischaemia/Reperfusion

**DOI:** 10.3390/biomedicines10050988

**Published:** 2022-04-25

**Authors:** Podsawee Mongkolpathumrat, Anusak Kijtawornrat, Eukote Suwan, Sasimanas Unajak, Aussara Panya, Tonapha Pusadee, Sarawut Kumphune

**Affiliations:** 1 Graduate Programs in Biomedical Sciences, Faculty of Allied Health Sciences, Naresuan University, Phitsanulok 65000, Thailand; podsaweem61@nu.ac.th; 2 Integrative Biomedical Research Unit (IBRU), Faculty of Allied Health Sciences, Naresuan University, Phitsanulok 65000, Thailand; 3 Biomedical Engineering Institute (BMEI), Chiang Mai University, Chiang Mai 50200, Thailand; 4 Department of Physiology, Faculty of Veterinary Science, Chulalongkorn University, Bangkok 10330, Thailand; anusak.k@chula.ac.th; 5 Department of Veterinary Technology, Faculty of Veterinary Technology, Kasetsart University, Bangkok 10900, Thailand; cvteks@ku.ac.th; 6 Department of Biochemistry, Faculty of Science, Kasetsart University, Bangkok 10900, Thailand; sasimanas.u@ku.ac.th; 7 Department of Biology, Faculty of Science, Chiang Mai University, Chiang Mai 50200, Thailand; aussara.pan@cmu.ac.th; 8 Department of Plant and Soil Science, Faculty of Agriculture, Chiang Mai University, Chiang Mai 50200, Thailand; tonapha.p@cmu.ac.th

**Keywords:** myocardial ischaemia/reperfusion injury, SLPI, cardioprotection, protease activity, anti-protease deficient

## Abstract

Inhibition of proteases shows therapeutic potential. Our previous studies demonstrated the cardioprotection by the Secretory Leukocyte Protease Inhibitor (SLPI) against myocardial ischaemia/reperfusion (I/R) injury. However, it is unclear whether the cardioprotective effect of SLPI seen in our previous works is due to the inhibition of protease enzymes. Several studies demonstrate that the anti-protease independent activity of SLPI could provide therapeutic benefits. Here, we show for the first time that recombinant protein of anti-protease deficient mutant SLPI (L72K, M73G, L74G) (mt-SLPI) could significantly reduce cell death and intracellular reactive oxygen species (ROS) production against an in vitro simulated I/R injury. Furthermore, post-ischaemic treatment of mt-SLPI is found to significantly reduce infarct size and cardiac biomarkers lactate dehydrogenase (LDH) and creatine kinase-MB (CK-MB) activity, improve cardiac functions, attenuate I/R induced-p38 MAPK phosphorylation, and reduce apoptotic regulatory protein levels, including Bax, cleaved-Caspase-3 and total Capase-8, in rats subjected to an in vivo I/R injury. Additionally, the beneficial effect of mt-SLPI was not significantly different from the wildtype (wt-SLPI). In summary, SLPI could provide cardioprotection without anti-protease activity, which could be more clinically beneficial in terms of providing cardioprotection without interfering with basal serine protease activity.

## 1. Introduction

Secretory leukocyte protease inhibitor (SLPI) is an 11.7 kDa cationic protein categorised in the serine protease inhibitor family [1]. It has been shown that SLPI is mainly expressed in several mucosal tissues, including nasal, bronchial, salivary, tear, cervical, and seminal secretions [2]. Various protease enzymes can be inhibited by SLPI, such as neutrophil elastase (NE), cathepsin G, trypsin, chymotrypsin, chymase, and tryptase [1,2]. In addition to inhibiting the protease enzyme, SLPI also demonstrates anti-bacterial [3], anti-viral [4], anti-inflammatory [5], anti-apoptosis [6], and anti-proliferative effects [7]. Our previous studies also indicated the cardio- and vasculo-protective effects of SLPI against myocardial ischaemia/reperfusion (I/R) injury, in both in vitro [8,9,10,11] and ex vivo [12,13] models. Recently, an in vivo post-ischaemic treatment of recombinant human SLPI (rhSLPI) also potentially reduced infarct size and improved cardiac function in an in vivo model of myocardial I/R injury [14]. Although the cardioprotective effect of SLPI is proven, it remains unclear whether the cardioprotective effect of SLPI seen in our previous works is due to the inhibition of protease enzymes. If so, anti-protease deficient SLPI could be required to answer the research question. Another concern is the adverse effects of the protease inhibitor treatment. Although it has been established that inhibition of proteases could demonstrate therapeutic applications, several adverse effects of protease inhibitor treatment have also been reported [15,16]. Therefore, anti-protease deficient mutant SLPI has been developed to investigate if the cardioprotection effect is dependent upon its anti-protease activity and whether it could be an alternative form of SLPI that may provide beneficial affects with fewer unexpected drug reactions.

Systematic residue alterations reveal that SLPI’s inhibitory activity against serine proteases is mediated by leucine-72 (Leu72), which is located within a putative inhibitory loop region that extends out of domain 2 [17]. Meanwhile, the C-terminal mediates direct protease inhibition and it has been suggested that the N-terminal domain stabilises any resultant complex formed from an SLPI/neutrophil elastase interaction [18]. Mutation at Leu72 together with two other amino acids in the inhibitory loop region, Met73 and Leu74, could cause SLPI to lose its ability to inhibit protease enzymes [19]. Previous studies reported that the beneficial effect of SLPI is independent of its anti-protease activity. For instance, SLPI activity to suppress macrophage response to bacterial lipopolysaccharide (LPS), which is known to be independent of its anti-protease activity [20]. Moreover, anti-protease deficient variants of SLPI could also inhibit TNF-α-induced apoptosis via attenuation of caspase-3 activation and DNA degradation [6]. Therefore, it could be concluded that the anti-apoptotic activity of SLPI is independent of its anti-protease activity.

In the present study, we demonstrate for the first time that anti-protease deficient mutant SLPI (L72K, M73G, L74G) reduced myocardial I/R injury with in vitro hypoxia/reoxygenation in cardiac cells while post-ischaemic systemic treatment of anti-protease deficient mutant SLPI could reduce infarct size and improve cardiac function against an in vivo myocardial I/R injury.

## 2. Materials and Methods

### 2.1. Cell Culture

H9c2—rat cardiomyoblast cell line (ATCC-CRL1446) was purchased from the American Type Culture Collection (Manassas, VA, USA). Cell culture was performed according to product recommendation using Dulbecco’s Modified Eagle Medium (DMEM) supplement with 10% (*v*/*v*) foetal bovine serum (FBS; GIBCO, Grand Island, NY, USA), 5000 units/mL of penicillin/streptomycin (GIBCO, Grand Island, NY, USA). Cells were cultured at 37 °C under humidified atmosphere 5% carbon dioxide.

### 2.2. An In Vitro Hypoxia/Reoxygenation Induce Cardiac Cell Death

An in vitro Hypoxia/Reoxygenation (H/R) protocol was modified from a previous study [21]. The 1.0 × 10^4^ cells were seeded into 96 well plated for 24 h until 70–80% confluence. Pre-treatment of recombinant human SLPI (both wild type; wt-SLPI or mutant; mt-SLPI) was performed at an equal concentration of 1 μg/mL for 1 h. Hypoxia was performed by overlaying the culture well with 200 μL liquid paraffin and incubated at 37 °C for 1 h. At the end of the hypoxic period, the overlay liquid and culture medium was replaced by a completed medium and incubated at 37 °C for 3 h. The cell viability was read out by using 3-(4,5-dimethylthiazo-yl)-2,5-diphenyl-2-H-tetrazolium bromide (MTT, ThermoFisher Scientific, Waltham, MA, USA) cell survival assay.

### 2.3. Production of Recombinant Mutant Forms of SLPI (mt-SLPI) and Wildtype (wt-SLPI)

The mutant SLPI plasmid harbouring the amino acids substitutions, which is critical for protease inhibition Leu^72^Lys, Met^73^Gly, Leu^74^Gly, (L72K, M73G, L74G) was kindly provided by Professor Joanna Cichy, Jagiellonian University, Kraków, Poland. The cDNA encoding mutant SLPI was generated by polymerase chain reaction (PCR), using RNA isolated from human alveolar epithelial cells as a template, and the mutations were generated by site-directed mutagenesis [19]. The cDNA containing mutant SLPI from PCR reaction was inserted to pPIC9 expression vector (Life Technologies, Carlsbad, CA, USA) with the addition of a hexahistidine tag and an enterokinase cleavage site to its N terminus [19]. The mutant SLPI—pPIC9 was transformed into *Escherichia coli* TOP10. The transformants were screened using colony PCR, and the corrected plasmid was transformed into Pichia pastoris KM71H. The protein expression level was optimized according to the manufacturer’s instructions (Life Technologies, Carlsbad, CA, USA).

The wildtype recombinant human SLPI (wt-SLPI) protein was purchased from was purchased from Sino Biology Inc. (Beijing, China).

### 2.4. Purification of Anti-Protease Deficient Mutant SLPI

A single colony of mt-SLPI was inoculated in Yeast Extract-Peptone-Dextrose (YPD) broth supplemented with 100 µg/mL zeocin and incubated at 30 °C, 250 rpm for overnight. Then, the cell was harvested, re-suspended with Buffered Glycerol Complex Medium (BMGY) broth in a ratio of 1:10 (*v*/*v*), and incubated at 30 °C 250 rpm. After 16–18 h incubation, the cell was harvested and re-suspended to an OD_600_ of 10 in Buffered Methanol-complex Medium (BMMY) and further incubated at 30 °C, 250 rpm. The final concentration of 1% (*v*/*v*) methanol was added daily. After 7 days of incubation, the cell suspension was spin down, and the supernatant was collected and loaded onto nickel-nitrilotriacetic acid matrix (Ni^2+^—NTA) affinity chromatography (GE Healthcare, Uppsala, Sweden). The recombinant protein was eluted with a stepwise gradient of 50, 100, 150, 250, and 500 mM of imidazole in 50 mM Tris—HCL, pH 8.0, and 300 mM NaCl. The purified protein was confirmed by Western blot analysis using an anti-His tag antibody.

### 2.5. Assessment of Inhibitory Activities of Anti-Protease Deficient Mutant by Neutrophil Elastase Activity

The activity of neutrophil elastase (NE) was measured by using a neutrophil elastase activity kit purchased from Abcam (ab204730, Cambridge, UK). The neutrophil elastase was preincubated for 10 min with recombinant human SLPI (wild type; wt-SLPI) and anti-protease deficient mutants (mutant; mt-SLPI) at 2:1 [SLPI:NE] molar ratio, at 20 °C in 0.1 M Tris-HCl (pH 8.0) with 0.5 M NaCl for 1 h. The SLPI-NE mixture and mutant SLPI-NE mixture were added into the reaction mix containing the neutrophil elastase buffer and substrate, while standard NE was used as control. A reaction was incubated at 37 °C for 20 min. Then, the activity was measured with a fluorescence microplate reader at 380/500 nm (excitation/emission). The activity of neutrophil elastase was expressed as % relative fluorescence units (RFU) of standard NE control and subtracted with the sample background.

### 2.6. Determination of Cell Viability by MTT Assay

MTT (3-[4,5-dimethylthiazol-2-yl]-2,5-diphenyltetrazolium bromide) was purchased from Thermo Fisher scientific. At the end of the study protocol, the culture medium was removed, and 200 μL of 0.5 mg/mL MTT reagent was added and incubated for 4 h. After incubation, the dimethyl sulfoxide (DMSO) was added to solubilize the formazan crystal and subsequently determined the absorbance using a spectrophotometer at a wavelength of 490 nm [14].

### 2.7. Determination of Intracellular Reactive Oxygen Species (ROS) Production

The determination of intracellular reactive oxygen species (ROS) production was followed by the previous report [13] using 2′,7′-Dichlorofluorescin diacetate (DCFH—DA, Sigma, St. Louis, MO, USA). Briefly, cells were cultured in 96 well plates with DMEM. The culture medium was removed and replaced with 2 µM DCFH-DA in serum-free DMEM in a dark room for 24 h at 37 °C. After H/R protocol, the medium was removed and replaced with a completed DMEM with DCFH-DA and incubated at 37 °C for 1 h. The fluorescence intensity was measured by using an EnSpire^®^ Multimode Plate Reader (PerkinElmer, Waltham, MA, USA) with an excitation wavelength of 485 nm and an emission wavelength of 530 nm.

### 2.8. Experimental Animal Ethical Approval and Experimental Groups

All experimental protocols used in this study were approved by the committee of the Centre for Animal Research, Naresuan University (Protocol number: NU—AE620615) and Chulalongkorn University Laboratory Animal Centre (CULAC) (Protocol number: 1973025). Animals were housed at 22 °C ± 1 °C, 12:12 h light:dark cycle, at the Chulalongkorn University Laboratory Animal Centre (CULAC), Bangkok, Thailand.

**Figure 1:**
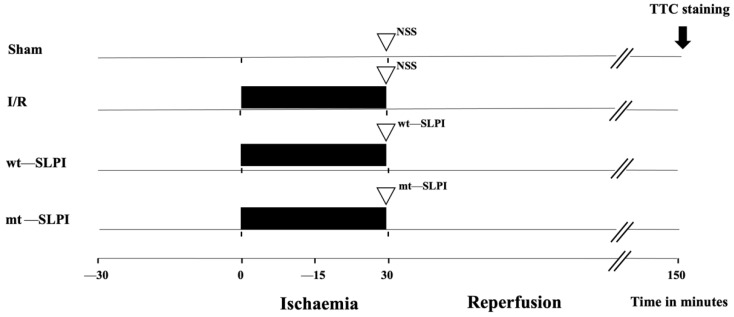
Schematic diagram of experimental protocol.

A total of 56 adult male Wistar rats (8 weeks old, 200–250 g) were purchased from Nomura Siam International, Bangkok, Thailand. Rats were divided into 4 groups (Figure 1) (*n* = 14/groups), and each group was divided into 2 subgroups, including 8 rats for infarct size and physiological study and 6 rats for biochemical analysis.

The group of the experimental protocol included the sham operation control group, where the surgery was performed without left anterior descending (LAD) coronary artery ligation in the sham group. In the I/R group, rats were subjected to LAD ligation for 30 min and reperfusion for 120 min. In treatment groups, 50 μg of recombinant human SLPI (wt-SLPI) or mutant rhSLPI (mt-SLPI) in 1 mL of phosphate buffer saline (PBS) was administered by intravenous injection at the onset of reperfusion.

### 2.9. Surgical Preparation of Myocardial I/R Model in Rats

The surgical procedure in this study was performed as described previously [14]. After rats were anesthetized by isoflurane inhalation, the tracheotomy, intubation, and ventilator connection (VentElite, Harvard apparatus, Holliston, MA, USA) were performed. The electrocardiogram (ECG) was set up for monitoring the alteration of the cardiac electrical signal. The left ventricular pressure (LVP) parameters were measured by using Mikro-Tip^®^ pressure catheter for rat (Millar, Houston, TX, USA) that cannulated through the carotid artery. Blood samples were collected from a fluid-filled catheter that cannulated at the jugular vein. In addition, the jugular vein canulation was also used for administering the wt-SLPI and mt-SLPI. Then, the left coronary artery was approached through the left thoracotomy. The left anterior descending coronary artery (LAD) was tied with 6-0 synthetic polypropylene suture to simulate ischaemia. The sham operation group underwent the same thoracotomy procedure and the other procedure except for the LAD ligation. The ischaemic condition was confirmed by ECG, showing ST-segment elevation and a faded whitish colour of the epicardium below the ligation. Myocardial ischaemia was induced for 30 min, followed by reperfusion for 120 min. The LVP parameters were recorded throughout the experimental period. The LVP parameters included end-diastolic pressure (EDP), end-systolic pressure (ESP), develop pressure (devP), and heart rate (HR). The contractility was measured by the dP/dt_max_ and the contractility index (CI). The relaxation indices were determined by the dP/dt_min_ and Tau. At the end of reperfusion period, the heart was rapidly excised, and then perfused with Evan blue and TTC staining for determining the infarct size.

### 2.10. Evaluation of Infarct Size and Area at Risk

The evaluation of infarction and area at risk was performed as described previously [14]. The area at risk (AAR) was assessed by perfusing with 2% (*w*/*v*) Evans blue. The hearts were sliced in 1 mm—thick slices and incubated with 1% (*w*/*v*) of 2,3,5-triphenyl tetrazolium chloride (TTC) at 37 °C for 10 min to determine the viable tissue and assessment of the infarct area. The heart sections were incubated with 10% (*v*/*v*) formalin before image analysis by using ImageJ software.

### 2.11. Determination of Serum Creatine Kinase (MB Isoenzyme) and Lactate Dehydrogenase (LDH) Activity

Blood collection was performed at the end of the animal protocol. The blood sample was centrifuged at 3000× *g* for 10 min at 4 °C. The serum was collected and stored at −80 °C until further investigation. Creatine kinase (MB isoenzyme) and lactate dehydrogenase (LDH) activity was determined and analysed by Cobas c 111 automate biochemistry analyser from Roche.

### 2.12. Tissue Homogenate and Protein Collection

Another set of experimental animals (*n* = 6 for each group) was subjected to I/R injury. After the surgery, the heart was excised immediately at the end of the experiment. The ventricular tissue was collected and snap-frozen in liquid nitrogen. The ventricular tissue (100 mg) was homogenised by using a hand homogeniser on ice. Tissue homogenate was then collected and centrifuged at 14,000 rpm for 10 min at 4 °C. The supernatant was collected and frozen for further experiment.

### 2.13. Western Blot Analysis

The heart homogenates protein was separated on 10 or 12% SDS-polyacrylamide gels and transferred into Immobilon-P membranes (Merck, Darmstadt, Germany). Membranes were incubated in 5% (*w*/*v*) dried skimmed milk powder in Tris—buffered saline (pH 7.4) containing 0.1% Triton X—100 (TBST) buffer for 1 h at room temperature. After that, membranes were probed overnight at 4 °C with the appropriate primary antibodies for phosphorylated p38 (Cell signalling Technology, Danvers, MA, USA), #9211), total-p38 (Cell signalling Technology, Danvers, MA, USA, #8690), Bax (Santa Cruz Biotechnology, Inc., Dallas, TX, USA, #sc—493), cleaved-caspase 3 (Cell signalling Technology, Danvers, MA, USA, #9661), and total—caspase-8 (Cell signalling Technology, Danvers, MA, USA, #9746) (All of primary antibodies were diluted at 1:1000 in 1% (*w*/*v*) skimmed milk + TBST buffer) Then, the membranes were washed and exposed for 1 h at room temperature to horseradish peroxidase-conjugated secondary antibody (goat anti-rabbit (Merck, Darmstadt, Germany, #AP132P) at 1:5000 in 1% (*w*/*v*) skimmed milk in TBST buffer. The signal was developed by exposure of the membranes with Affinity^®^ ECL Western Blot Kit, picogram Grade (Affinity Biosciences, Melbourne, Australia). The band intensity quantitation was captured and analysed by using ImageQuant™ LAS 500 (GE Healthcare, Chicago, IL, USA).

### 2.14. Statistical Analysis

All values were expressed as mean ± S.E.M. Data sets were analysed by One-way analysis of variance (ANOVA), and groups were compared using Tukey’s test. The statistical tests were performed using commercially available software (Lab chart Prism version 5). A *p*-value of less than 0.05 was considered statistically significant.

## 3. Results

### 3.1. The Neutrophil Elastase Activity of Wild-Type and Mutant SLPI on H9c2 Cells

**Figure 2:**
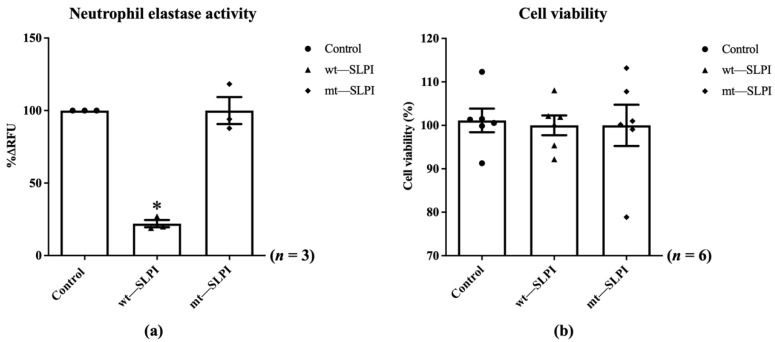
Determination of the enzymatic activity of neutrophil elastase in the presence of wild-type(wt) or mutant(mt) SLPI (**a**), cell viability after treated with wt-SLPI and mt-SLPI (**b**) (* *p* < 0.05 vs. control).

The enzymatic activity of neutrophil elastase in the presence of wild-type(wt) or mutant(mt) SLPI were measured by fluorometric technique. The results showed that the enzymatic activity of NE was significantly inhibited when preincubated with wt-SLPI to 22.0 ± 2.51%RFU when compared to that of the control (*p* < 0.05), whereas preincubated NE with mt-SLPI failed to inhibit the NE activity (Figure 2a). Then, both of wt-SLPI and mt-SLPI were treated to H9c2 and was determined for cellular toxicity. The results showed that there was no significant difference in cell viability when cells were exposed to both wt-SLPI and mt-SLPI in comparison to those of the control group (100 ± 2.44 and 100 ± 5.29 vs. 101.1 ± 2.03, *p* > 0.05) (Figure 2b).

### 3.2. Anti-Protease Deficient SLPI Reduced Cardiac Cell Injury and Intracellular ROS Production in Hypoxia/Reoxygenation (H/R) Condition

**Figure 3:**
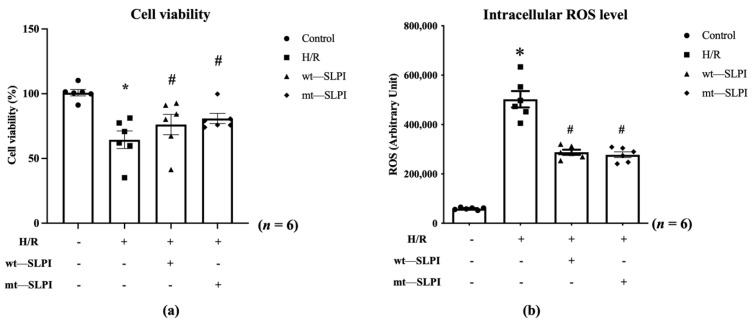
Determination of percentage of cell viability (**a**) and intracellular ROS (**b**) under H/R condition and treated with wt-SLPI and mt-SLPI (* *p* < 0.05 vs. control) (*^#^ p* < 0.05 vs. H/R).

An in vitro cardioprotective effect of mt-SLPI was performed against hypoxia/reoxygenation (H/R) conditions. The results showed that H/R conditions could significantly decrease cell viability when compared with the control group (63.67 ± 5.03 vs. 100.2 ± 1.94, *p* < 0.05). After being treated with mt-SLPI, the result showed that mt-SLPI significantly increased in cell viability when compared with the H/R group (80.89 ± 4.10 vs. 63.67 ± 5.03, *p* < 0.05), which was comparable to wt-SLPI (80.58 ± 4.35 vs. 63.67 ± 5.03, *p* < 0.05) (Figure 3a).

Intracellular ROS was significantly increase in H/R group when compared with control (H/R: 502,411 ± 32,944 vs. Control: 59,226 ± 1942 Arbitrary unit (A.U.), *p* < 0.05). Treatment with both of wt-SLPI and mt-SLPI could significantly reduce intracellular ROS level when compared with H/R group (wt-SLPI; 287,781 ± 10,312 and mt-SLPI; 277,509 ± 11,643 vs. Control; 502,411 ± 32,944 Arbitrary unit (A.U.), *p* < 0.05) (Figure 3b).

### 3.3. Anti-Protease Deficient SLPI Reduced Infarct Size Cardiac Biomarkers in an In Vivo I/R Injury

**Figure 4:**
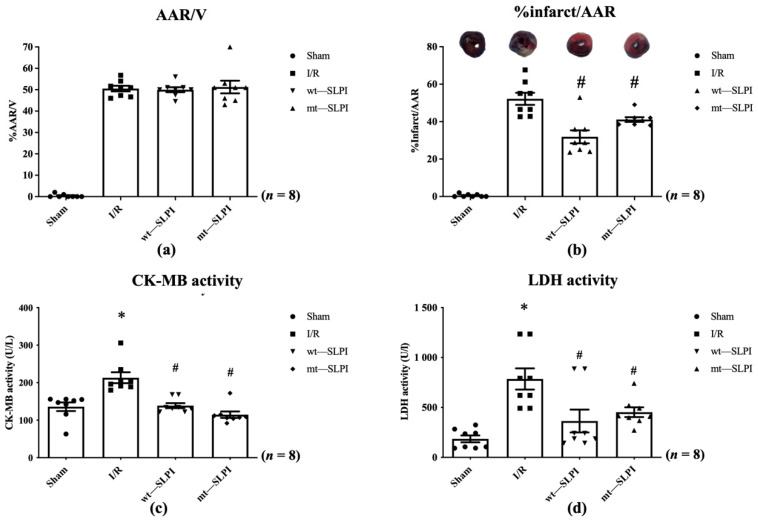
The percentage of area at risk (AAR) to ventricle volume (V) (**a**), percentage of infarct size to area at risk (**b**), Creatine kinase-MB isoenzyme (CK-MB) activity (**c**), and activity of Lactate Dehydrogenase (LDH) activity (**d**) (* *p* < 0.05 vs. sham) (^#^ *p* < 0.05 vs. I/R) (*n* = 8).

In this study, all animals survived throughout the experimental protocol period (0% mortality). At the end of reperfusion, the hearts were excised and measured for infarct size. The results showed that there was not a significant difference in % area at risk (AAR) in the I/R injury group and both treated groups (Figure 4a). Results in Figure 4b showed that infarct size in both of wt-SLPI and mt-SLPI treated groups was significantly lower than that of I/R group (31.75 ± 4.64, 41.60 ± 1.98 vs. 52.65 ± 4.23, *p* < 0.05).

The serum LDH and CK-MB activity were measured at the end of the study protocol. The results showed that CK-MB activity in I/R group was significantly increased when compared with the sham group (213.1 ± 14.48 vs. 135.9 ± 11.42 U/L), *p* < 0.05) (Figure 4c). Treatment with both wt-SLPI and mt-SLPI could significantly reduce CK-MB activity when compared with I/R group (138.8 ± 10.08 and 122.5 ± 7.34 vs. 213.1 ± 14.48 U/L), *p* < 0.05) (Figure 4c).

Similarly, there was a significant increase in LDH activity of the I/R group than the sham group (785.3 ± 162.1 vs. 187.8 ± 30.12 U/L, *p* < 0.05) (Figure 4d). Treatment group both of wt-SLPI and mt- SLPI was significantly reduced LDH activity than that of I/R group (364.5 ± 175.0 and 452.2 ± 43.43 vs. U/L), *p* < 0.05) (Figure 4d).

### 3.4. Effect of Anti-Protease Deficient SLPI Treatment on Cardiac Function Parameters in I/R Injury

The results of LVP were divided into three phases according to the surgery processes including baseline (30 min prior to LAD ligation), ischaemia (at the end of LAD ligation period), and reperfusion (at the end of reperfusion period).

At the baseline phase, all of the LVP parameters were not significantly different among groups (Table 1).

At the ischemic phase, there was a reduction in ESP, dP/dt_max_, devP and increased EDP, dP/dt_min_ and Tau/e in I/R, wt-SLPI, and mt-SLPI group when compared with the sham group (Table 2).

At the reperfusion phase, the I/R injury induced the reduction of ESP, dP/dt_max,_ devP and increase of EDP, dP/dt_min,_ and Tau/e when compared with the sham group. After treatment with wt-SLPI, the results showed wt-SLPI could increase ESP, dP/dt_max,_ devP and reduced EDP, dP/dt_min,_ and Tau/e when compared with the I/R group (Table 3). Whereas mt-SLPI treatment could reduce only the EDP parameter when compared with the I/R group.

### 3.5. Effect of Anti-Protease Deficient SLPI on p38 MAPK Phopshorylation and Apoptosis Regulatory Molecules

**Figure 5:**
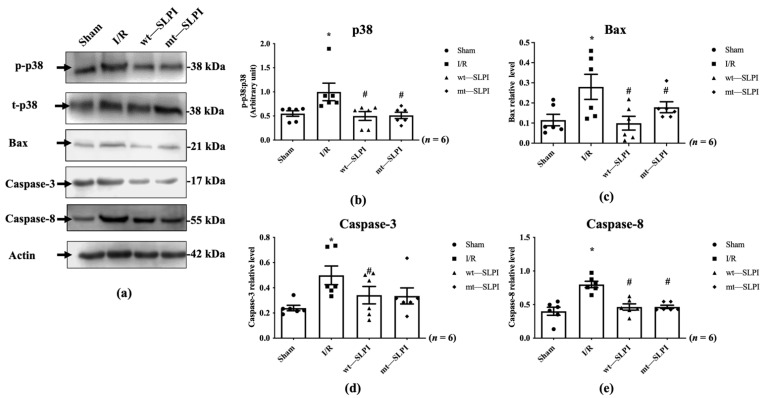
Determination of the apoptotic regulatory signalling protein level in MI rat by Western blot analysis (**a**). The quantitative analysis for the expression was presented for p38 MAPK phosphorylation (**b**), Bax (**c**), cleaved Caspase-3 (**d**) and total Caspase-8 (**e**) (* *p* < 0.05 vs. sham) (^#^ *p* < 0.05 vs. I/R) (*n* = 6).

The signal transduction that responds to I/R in the presence and absence of wt-SLPI and mt-SLPI at the onset of reperfusion was determined by Western blot analysis. The results showed that there was a significant activation of p38 MAPK in the I/R group when compared with the sham group (1.07 ± 0.18 vs. 0.50 ± 0.07, *p* < 0.05) (Figure 5a). Treatment of both wt-SLPI and mt-SLPI at the onset of reperfusion could significantly reduce phosphorylated p38 MAPK when compared with the I/R group (0.49 ± 0.09 and 0.50 ± 0.09 vs. 1.07 ± 0.18, *p* < 0.05). Similar results were observed in the apoptotic regulatory pathway, including Bax, cleaved caspase-3, and total caspase-8. The results showed that I/R injury significantly increased pro-apoptotic proteins level when compared with the sham group (Bax: 0.28 ± 0.04 vs. 0.11 ± 0.02, cleaved caspase-3: 0.49 ± 0.06 vs. 0.24 ± 0.02, total caspase-8: 0.79 ± 0.03 vs. 0.39 ± 0.03, *p* < 0.05) (Figure 5b–d). Treatment of both wt-SLPI and mt-SLPI significantly reduced the pro-apoptotic proteins level when compared with the I/R group (Bax: 0.10 ± 0.02 and 0.15 ± 0.01 vs. 0.28 ± 0.04, cleaved caspase-3: 0.32 ± 0.04 and 0.35 ± 0.09 vs. 0.49 ± 0.06 total caspase-8: 0.46 ± 0.03 and 0.46 ± 0.03 vs. 0.79 ± 0.03, *p* < 0.05) (Figure 5b–d).

## 4. Discussion

This is the first study to show the cardioprotective effect of anti-protease deficient SLPI in both an in vitro hypoxia/reoxygenation in cardiac cells and administered as post-ischaemic treatment in a rat model subjected to an in vivo myocardial I/R injury. The major findings are that the mutant form SLPI (mt-SLPI) containing triple substitutions of amino acids critical for SLPI’s anti-protease activity (L72K, M73G, L74G) could reduce cell death, infarct size, cardiac injury markers, and improved cardiac functions by attenuation of intracellular signalling molecules involved in cell death, including p38 MAPK, Bax, and Caspase-8.

In a myocardial ischaemia/reperfusion injury, the level of protease enzymes activity increases for both intracellular and extracellular cardiac cells in order to maintain homeostasis of the cellular structure by degrading damaged proteins [22]. Normally, protease enzymes play a crucial role in producing and degrading proteins to recycle dysfunctional and damaged proteins in the cell [23]. Myocardial injury during ischaemia contributes to the accumulated activated neutrophil in the injury area. The accumulation of activated neutrophils leads to tissue injury due to the release of cytotoxic cytokines, including inflammatory cytokines and proteases enzymes, which eventually result in cell death [24]. Moreover, after reperfusion, these are attributed to more activated reactive oxygen species (ROS), inflammatory cytokines, and accumulation of neutrophils [13,25]. Increasing ROS and inflammatory cytokines levels triggered the recruitment and infiltration of neutrophils and mast cells, which released serine proteases, including cathepsin, calpain, and matrix metalloproteinase (MMP), which are key mediators involved in cardiovascular diseases progression [26]. Post-MI patients present an increased level of protease enzyme in serum, which is related to growth factor levels and connected to fibrosis development [27,28]. Therefore, the protease enzyme plays a crucial role in the development of cardiovascular diseases and their progression. Moreover, an imbalance of protease activity as well as protease inhibitors in the cell contribute to cardiac cell injury and the changing the structure of cardiac tissue, such as cardiac remodelling or cardiac hypertrophy [22]. Therefore, the inhibition of serine protease enzymes has therapeutic potential and applications to prevent the progression of cardiac remodelling or heart failure after myocardial infarction.

The protease enzyme inhibitory strategy has been shown to offer therapeutic potential to reduce cardiac cell death, injury, and remodelling in several study models [29,30,31,32], including the authors’ previous study on SLPI. SLPI have been proved to reduce cardiac cell death in ischaemia/reperfusion injury, which is believed to reduce reactive oxygen species, attenuate p38 MAPK activation that predominantly regulates cellular injury in myocardial I/R, and enhance the Akt cell survival pathway, which subsequently attenuates the apoptotic regulatory pathways [8,9,10,11,12,13,14]. This evidence suggests that inhibition of protease activity could be a major contributing mechanism that explains the therapeutic potential of SLPI. Yet, it is well known that serine proteases are crucial biological effectors that play several important roles in biological and physiological processes, including blood coagulation, fibrinolysis, and complement activation [33]. It has been reported that patients who receive protease inhibitors could develop several side effects such as hyperglycaemia, haemolytic anaemia, and spontaneous bleeding in haemophiliac patients [15], in addition to metabolic side effects on cholesterol and triglycerides [16]. It is, therefore, important that therapeutic protease inhibitors are used carefully, and there is sufficient awareness of potential adverse drug effects. This prompted an idea on whether SLPI could potentially be used as a drug, a major concern which could also focus on post-treatment adverse drug effects. Therefore, using SLPI without anti-protease activity could be an alternative option.

**Figure 6:**
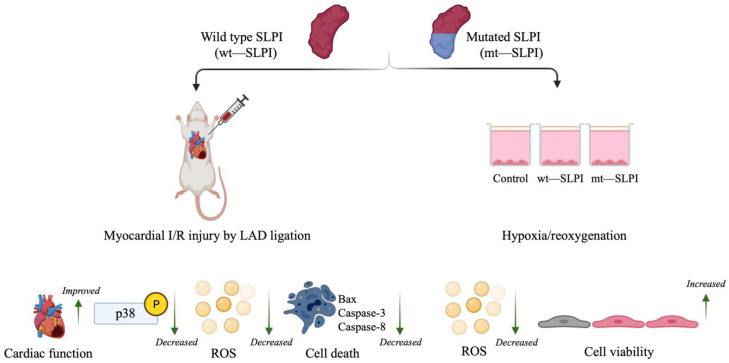
A schematic diagram of the major findings in this study. Both type of wild type and mutant (anti-protease deficient) SLPI could improve cell viability, reduce infarct size, and improve cardiac function. These could associate with attenuation of p38 MAPK activation, intracellular ROS production, and activation of apoptotic regulatory signalling proteins, Bax, Caspase-3 and Caspase-8.

It is well known that the central role of the protease inhibitory effect of SLPI is located at Leu72-Met73 residues [3]. However, the mechanism of the direct effects of SLPI as well as intracellular signal transduction downstream of SLPI stimulation remains unclear. Mutation of the critical amino acids that govern SLPI anti-protease activity has been implemented, and results have been interpreted as losing the ability to inhibit serine protease, which could not affect the beneficial effect of SLPI, particularly for anti-HIV infection and anti-inflammation [4,19]. Nonetheless, anti-protease activity deficient SLPI has never been investigated with cardiovascular diseases, particularly myocardial ischaemia/reperfusion injury. The present study demonstrates the first evidence with the cardiovascular model that anti-protease activity deficient SLPI treatment in the post-ischaemic condition could reduce infarct size, cardiac biomarkers, and inflammatory cytokines in addition to improving cardiac function (Figure 6).

Anti-protease deficient SLPI (L72K, M73G, L74G) has been generated and studies conducted with an immunological study model [19]. The potential mechanism of anti-protease deficient SLPI is intracellular interaction and competing binding of SLPI to p65, which prevents the activation of NF-κB, resulting in the reduction of TNF-α and IL-8 production [34]. In addition, SLPI binding partners have been identified; association of SLPI to a calcium-dependent phospholipid-binding membrane -Annexin A2 (AnxA2) enhanced AnxA2 phosphorylation [35], which consequently mediates the Akt-survival pathway activation [36]. Akt is one of the survival kinases that have an important role in cell survival [37]. It has been shown that treatment of SLPI also activates Akt phosphorylation and is related to a reduction of cellular injury in myocardial I/R injury [12,13,14]. In the current study, p38 MAPK activation related to the apoptotic regulatory pathway was demonstrated. However, the Akt phosphorylation should also be determined to strengthen the anti-apoptotic activity of SLPI. Another interesting binding partner is phospholipid cellular receptors-scramblases 1 and 4, which resulted in the inhibition of HIV-1 infection [38]. Scramblases can bind to onzin, the c-Myc transcription factor. The Association of scramblases and onzin could interact with Akt and Mdm2, inhibit p53, and lead to inhibiting cell apoptosis [39]. This indicates that SLPI could directly affect signalling without anti-protease activity by binding to molecules involved in the survival pathway.

The findings from the present study provide evidence that mt-SLPI and wt-SLPI appear to not be significantly different in several parameters. This suggests that the anti-protease activity of SLPI may not be required for cardioprotection since mt-SLPI demonstrates a cardioprotective effect similar to wt-SLPI. However, the results of %infarct size/%AAR (Figure 4b) show that wt-SLPI tends to provide greater protection than mt-SLPI. This is also related to the cardiac function presented in Table 3, in which most of the ventricular pressure parameters are not significantly different between wt- and mt-SLPI, except for the EDP. From this, it can be concluded that inhibition of protease could also be essential and the direct effect of SLPI could be orchestrated to maximise cardioprotection.

Serine proteases constitute the most numerous groups of proteases, accounting for 40%, and they are prevalent in many physiological functions, including both normal and disease-related functions [40,41,42]. Inhibition of serine protease also interferes with physiological homeostasis. The anti-protease independent effect of SLPI in this current study, specifically myocardial I/R injury, could highlight the “direct effect” of SLPI on cardioprotection.

Implementation of anti-protease deficient recombinant human SLPI could be more clinically beneficial in terms of providing cardioprotection without interfering with basal serine protease activity. Therefore, further investigation of anti-protease deficient recombinant human SLPI in a more clinically related study should be performed, since the findings of the present study suggest a clinical study would provide highly useful information. Since the half-life of SLPI after administration is short (approximately 60 min in circulation) [43], any strategies to prolong the half-life of SLPI, increase its stability, or offer a more efficient delivery method require investigation. A previous study using a chimeric protein of SLPI-Cementoin fusion protein found that it could successfully enhance the biological activity of SLPI [44]. Therefore, the chimeric of anti-protease deficient SLPI-Cementoin has an interesting potential to enhance the physiological activity of anti-protease deficient recombinant human SLPI.

Some limitations of the present study must be discussed and highlighted. The consequences of I/R injury included both cell injury in addition to cellular inflammation and immune responses, which are known to be involved in disease progression [45]. The inflammatory cytokines level, leukocytes infiltration, and the response of protease enzymes secreted from infiltrate leukocytes in the presence of mutant SLPI treatment should be investigated. Moreover, determination of an in situ protease inhibitory activity in cardiac tissue of both having wt-SLPI and mt-SLPI would also provide the explanation on tissue injury and protease activity and could also elucidate if the cardioprotective effect of SLPI require its anti-protease activity. As discussed previously concerning the direct effect of SLPI in association with binding partners such as Annexin A2, scramblases 1 and 4, further studies should prove the association between SLPI and those candidates’ binding partners in myocardial I/R injury, which would be challenging. This future research would provide greater mechanistic insight into the cardioprotective effect of SLPI.

## 5. Conclusions

Our findings show for the first time that treatment of anti-protease deficient recombinant human SLPI protein could reduce cardiac cell injury in an in vitro hypoxia/reoxygenation, reduce infarct size, and improve the cardiac function of rats subjected to an in vivo myocardial I/R injury. The results suggest that the anti-protease activity of SLPI may not be required, which could potentially be a direct effect of SLPI on cardioprotection.

## Figures and Tables

**Table 1:** The baseline left ventricular pressure (LVP) parameters.

Parameters	Group			
	Sham (*n* = 8)	I/R (*n* = 8)	wt-SLPI (*n* = 8)	mt-SLPI (*n* = 8)
EDP (mmHg)	5.07 ± 0.73	4.37 ± 0.12	4.70 ± 0.21	4.23 ± 0.20
ESP (mmHg)	118.7 ± 5.63	112.7 ± 7.15	122.2 ± 18.42	110.6 ± 6.17
dP/dt_max_ (mmHg/s)	4958 ± 328.0	4778 ± 367.30	5709 ± 226.0	4973 ± 662.6
CI	101.5 ± 2.47	104.4 ± 6.49	105.9 ± 3.29	103.6 ± 6.28
dP/dt_min_ (mmHg/s)	−4668 ± 295.0	−4884 ± 466.7	−4935 ±444.2	−4938 ± 336.0
Tau/e (ms)	10.32 ± 0.23	10.63 ± 0.24	8.66 ± 0.35	10.17 ± 0.11
devP (mmHg)	113.8 ± 4.41	116.2 ± 7.92	119.1 ± 11.59	110 ± 6.38
HR (bpm)	447 ± 11.13	438 ± 21.36	450 ± 7.02	450 ± 16.01

**Table 2:** The left ventricular pressure (LVP) parameters of ischemic.

Parameters	Group			
	Sham (*n* = 8)	I/R (*n* = 8)	wt-SLPI (*n* = 8)	mt-SLPI (*n* = 8)
EDP (mmHg)	5.36 ± 0.25	8.08 ± 0.99 *	7.29 ± 0.11 *	6.72 ± 0.65 *
ESP (mmHg)	118.1 ± 7.12	90.83 ± 5.02 *	89.65 ± 11.83 *	98.56 ± 7.89 *
dP/dt_max_ (mmHg/s)	5517 ± 810.0	3547 ± 272.1 *	4090 ± 215.2 *	3395 ± 550.1 *
CI	99.86 ± 6.67	96.03 ± 4.80	107.8 ± 10.72	99.14 ± 4.15
dP/dt_min_ (mmHg/s)	−4267 ± 124.3	−3108 ± 61.71 *	−3162 ± 612.1 *	−3127 ± 486.5 *
Tau/e (ms)	9.71 ± 0.35	11.77 ± 0.64 *	10.03 ± 0.67	11.76 ± 0.25 *
devP (mmHg)	113 ± 6.84	83.47 ± 7.72 *	94.85 ± 6.87 *	90.84 ± 5.41 *
HR (bpm)	444 ±16.51	423 ± 20.68	432 ± 7.72	447 ± 9.24

*p < 0.05 vs. sham.

**Table 3:** The left ventricular pressure (LVP) parameters of the reperfusion phase.

Parameters	Group			
	Sham (*n* = 8)	I/R (*n* = 8)	wt-SLPI (*n* = 8)	mt-SLPI (*n* = 8)
EDP (mmHg)	6.20 ± 0.70	7.66 ± 0.06 *	4.42 ± 0.49 ^#^	5.50 ± 0.12 ^#^
ESP (mmHg)	109.7 ± 7.41	74.21 ± 7.09 *	94.47 ± 5.56 ^#^	79.58 ± 2.87
dP/dt_max_ (mmHg/s)	4872 ± 541.8	2688 ± 419.7 *	4437 ± 484.6 ^#^	3390 ± 338.1
CI	95.09 ± 6.07	80.05 ± 9.01	91.06 ± 11.58	93.65 ± 3.33
dP/dt_min_ (mmHg/s)	−5330 ± 510.1	−2252 ± 477.3 *	−3854 ± 293 ^#^	−2922 ± 292.5
Tau/e (ms)	10.97 ± 0.64	15.94 ± 4.40 *	8.131 ± 1.58 ^#^	12.57 ± 0.40
devP (mmHg)	106.6 ± 5.04	76.47 ± 8.06 *	101.1 ± 4.98 ^#^	86.59 ± 6.40
HR (bpm)	425 ± 18.01	411 ± 26.03	417 ± 10.07	411 ± 10.92

*p < 0.05 vs. sham, # *p* < 0.05 vs. I/R.

## Data Availability

The data presented in this study are available on request from the corresponding author.
